# Evaluation of ChatGPT-4 as an Online Outpatient Assistant in Puerperal Mastitis Management: Content Analysis of an Observational Study

**DOI:** 10.2196/68980

**Published:** 2025-07-24

**Authors:** Fatih Dolu, Oğuzhan Fatih Ay, Aydın Hakan Kupeli, Enes Karademir, Muhammed Huseyin Büyükavcı

**Affiliations:** 1 Department of Surgical Oncology, Kahramanmaras Necip Fazıl City Hospital, Kahramanmaras, Turkey; 2 Department of General Surgery, Kahramanmaras Necip Fazıl City Hospital, Merkez, Erkenez Mh., Recep Tayyip Erdoğan Bulvarı 12. KmKahramanmaras, 46050, Turkey, 90 3442282800; 3 Department of General Surgery, Bursa Yuksek Ihtisas Egitim Ve Arastirma Hastanesi, Bursa, Turkey

**Keywords:** puerperal mastitis, Artificial intelligence, ChatGPT, response quality, patient safety, DISCERN score, Flesch-Kincaid readability, treatment adherence, general surgery outpatient clinics, AI

## Abstract

**Background:**

The integration of artificial intelligence (AI) into clinical workflows holds promise for enhancing outpatient decision-making and patient education. ChatGPT, a large language model developed by OpenAI, has gained attention for its potential to support both clinicians and patients. However, its performance in the outpatient setting of general surgery remains underexplored.

**Objective:**

This study aimed to evaluate whether ChatGPT-4 can function as a virtual outpatient assistant in the management of puerperal mastitis by assessing the accuracy, clarity, and clinical safety of its responses to frequently asked patient questions in Turkish.

**Methods:**

Fifteen questions about puerperal mastitis were sourced from public health care websites and online forums. These questions were categorized into general information (n=2), symptoms and diagnosis (n=6), treatment (n=2), and prognosis (n=5). Each question was entered into ChatGPT-4 (September 3, 2024), and a single Turkish-language response was obtained. The responses were evaluated by a panel consisting of 3 board-certified general surgeons and 2 general surgery residents, using five criteria: sufficient length, patient-understandable language, accuracy, adherence to current guidelines, and patient safety. Quantitative metrics included the DISCERN score, Flesch-Kincaid readability score, and inter-rater reliability assessed using the intraclass correlation coefficient (ICC).

**Results:**

A total of 15 questions were evaluated. ChatGPT’s responses were rated as “excellent” overall by the evaluators, with higher scores observed for treatment- and prognosis-related questions. A statistically significant difference was found in DISCERN scores across question types (*P*=.01), with treatment and prognosis questions receiving higher ratings. In contrast, no significant differences were detected in evaluator-based ratings (sufficient length, understandability, accuracy, guideline compliance, and patient safety), *JAMA* benchmark scores, or Flesch-Kincaid readability levels (*P*>.05 for all). Interrater agreement was good across all evaluation parameters (ICC=0.772); however, agreement varied when assessed by individual criteria. Correlation analyses revealed no significant overall associations between subjective ratings and objective quality measures, although a strong positive correlation between literature compliance and patient safety was identified for one question (*r*=0.968, *P*<.001).

**Conclusions:**

ChatGPT demonstrated adequate capability in providing information on puerperal mastitis, particularly for treatment and prognosis. However, evaluator variability and the subjective nature of assessments highlight the need for further optimization of AI tools. Future research should emphasize iterative questioning and dynamic updates to AI knowledge bases to enhance reliability and accessibility.

## Introduction

Puerperal mastitis, also known as lactation mastitis, is a disorder that affects approximately 30% of breastfeeding women and may vary from mild inflammation to bacterial abscess formation [1]. This disease, which adversely affects breastfeeding, is monitored by the World Health Organization (WHO) and represents a substantial proportion of outpatient clinic applications in general surgery [2,3].

Artificial intelligence (AI)-based systems are currently regarded as capable of enhancing the workflow and aiding the decision-making process [4]. The health care system, particularly in outpatient management, aims to alleviate the patient burden and thereby enhance patient satisfaction [5]. Recent studies have shown that ChatGPT is capable of providing accurate differential diagnoses in 93.3% of common clinical scenarios, and its responses have achieved high accuracy in fields such as retinal diseases, gynecology, and hepatic disorders. These findings underscore the expanding role of AI-powered tools in enhancing diagnostic reasoning and clinical communication across a wide spectrum of medical disciplines [6-8]. In the future, AI is expected to progress as a conduit between clinical practice and data, thereby enhancing patient care and operations [9].

This study aimed to evaluate whether ChatGPT-4 could function as a virtual outpatient assistant in the management of puerperal mastitis. We assessed the content of its responses in Turkish to commonly asked patient questions, focusing on their adequacy, clarity, adherence to medical literature, and potential safety implications.

## Methods

### Study Design and Hypotheses

In this study, we aimed to evaluate whether ChatGPT can function as a competent assistant in general surgery outpatient settings by managing frequently encountered clinical conditions such as puerperal mastitis. The rationale behind this study is based on the dual expectation from ChatGPT: to provide clinically reliable information grounded in current medical literature and to effectively communicate this information in a clear, understandable manner suitable for patient interaction.

ChatGPT-4 was selected as the AI model for evaluation due to its widespread adoption in recent health-related studies and its extensive documentation in the current literature. Using ChatGPT-4 allowed for comparability with prior research, ensuring consistency and alignment with similar investigations examining large language models’ (LLMs’) performance in medical information delivery.

Our study was guided by three hypotheses: (1) the responses provided by ChatGPT regarding puerperal mastitis are adequate for daily clinical practice, (2) the quality of responses varies depending on the context and category of the question, (3) using language that is easier to understand may reduce adherence to current clinical literature and accuracy, thereby potentially compromising patient safety.

Ethics committee approval was not sought for this study, as no clinical or personal information about patients was used. However, preliminary permission was obtained from the affiliated hospital.

### Question Identification

Fifteen of the most frequently asked and clinically relevant questions about puerperal mastitis were identified through a review of publicly available health care websites, patient forums, and social media platforms. The decision to include 15 questions was based on a review of similar studies in the literature that used a comparable sample size to allow for focused qualitative and quantitative evaluation. This number was deemed sufficient to simulate a realistic outpatient consultation experience and to provide variability across clinical themes.

The questions were categorized to reflect the breadth of clinical concerns typically encountered in general surgery outpatient settings. These categories were general information (n=2), symptoms and diagnosis (n=6), treatment (n=2), and prognosis (n=5). Categorization aimed to allow structured evaluation across different domains of clinical relevance.

To reflect the linguistic and cultural context of our primary patient population, all questions were written in Turkish. The questions were then input into ChatGPT-40 mini, an AI chatbot, on September 3, 2024. A newly created user account was used to ensure that no prior interactions influenced the generated responses. The system was accessed under a standard free-tier usage scenario, without premium settings, plugins, or enterprise features enabled. This setup was intended to simulate how a general user or outpatient might realistically interact with the tool. Each question was entered only once, without any follow-up or clarification prompts. Responses were recorded without modification (refer to Multimedia Appendix 1 and Multimedia Appendix 2).

### Response Evaluation

The responses generated by ChatGPT were evaluated by a panel of 5 medical professionals, comprising 3 board-certified general surgeons (OFA, FD, and AHK) and 2 general surgery residents (MHB and EK). Detailed evaluation scores and comparative analyses are provided in Multimedia Appendix 3 and Multimedia Appendix 4.

Each response was independently scored using a 5-point Likert scale (1=poor and 5=excellent) according to the following 5 criteria: (1) sufficient length, (2) use of language understandable to the patient, (3) accuracy in answering the inquiry, (4) compliance with the most recent clinical guidelines, (5) patient safety. These criteria were selected based on methodologies used in similar studies within the literature that evaluated AI-based responses in medical contexts. The aim was to holistically capture both the technical validity and the patient communication aspects of the responses.

Based on total scores, responses were classified as (1) 5‐10: inadequate, (2) 11‐15: average, (3) 16‐20: adequate, and (4) 21‐25: excellent.

### Objective Assessment Tools

To further ensure objectivity and align with methodologies frequently used in similar literature evaluating AI-generated medical information, we used three standardized tools:

#### DISCERN Instrument

DISCERN is a standardized and validated tool developed by the University of Oxford for assessing the quality of consumer health information, particularly regarding treatment choices. Widely cited in the evaluation of online medical content, it consists of 15 questions that evaluate the publication’s reliability, objectivity, and clarity in describing treatment options. Each item is rated on a 5-point scale. Total scores categorize information quality as very poor (<27), poor (27-38), fair (39-50), good (51-62), and excellent (63-75) [10].

#### Journal of the American Medical Association Benchmark Criteria

Published by the *Journal of the American Medical Association* (*JAMA*), this benchmark is a qualitative framework used to assess the credibility of online health information. It includes four domains: authorship (clearly identified sources), attribution (references to content sources), disclosure (ownership and conflicts of interest), and currency (dates of publication or last update). In our study, the *JAMA* benchmark score was found to be zero, which reflects an inherent limitation of ChatGPT’s output—it does not include cited authors, publication dates, or institutional disclosure information in its responses. This has similarly been observed in other studies evaluating AI-generated health content and is a recognized limitation of current language models [11].

#### Flesch-Kincaid Grade Level Test

This tool was used to assess the complexity of language in the ChatGPT responses. The readability score was calculated using the following formula: Grade Level=(0.39× Total Sentences/Total Words)+(11.8× Total Words/Total Syllables)−15.59 to assess the complexity of language used by ChatGPT [12]. This assessment evaluates text understanding and target audience appropriateness; scores ranging from 0 to 5 indicate primary school level, 6 to 8 indicate secondary school level, 9 to 12 indicate high school level, and 13 and above indicate university level.

### Statistical Analysis

The normality of the data distribution was examined using the Shapiro-Wilk test. For the comparison of evaluator scores across more than 2 independent groups, one-way ANOVA and least significant difference multiple comparison tests were used for features showing normal distribution, while the Kruskal-Wallis test and all-pairwise multiple comparison tests were used for non-normally distributed features. Interobserver agreement among the 5 doctors for each parameter was measured using the intraclass correlation coefficient (ICC) (single measure and two-way random model). ICC values were classified as follows: “excellent” (above 0.90), “good” (0.75‐0.90), “moderate” (0.50‐0.75), and “poor” (less than 0.50) [13]. The relationships between the question scales were analyzed using the Spearman correlation coefficient. A correlation coefficient between 0.8 and 1 indicates a very strong relationship, values between 0.6 and 0.8 represent a strong relationship, values between 0.4 and 0.6 indicate a moderate relationship, and values between 0.2 and 0.4 signify a weak relationship. Differences between scores within the questions were examined using the Friedman test, with post-hoc tests corrected using the Bonferroni method. Descriptive statistics are presented as mean (SD) for numerical variables, and as counts and percentages for categorical variables. Statistical analyses were performed using SPSS for Windows (version 24.0; IBM), and statistical significance was set at *P*<.05.

### Ethical Considerations

This study did not involve human participants, identifiable personal data, clinical interventions, or medical record review; therefore, approval from an institutional review board or ethics committee was not required. In accordance with institutional and national guidelines governing noninterventional research, studies based solely on publicly available, nonidentifiable content—such as online health information and AI-generated responses—are exempt from ethics committee review. Nevertheless, prior administrative permission was obtained from Kahramanmaras Necip Fazıl City Hospital before the commencement of the study.

## Results

Differences in scores were analyzed according to the clinical categories of the questions and the evaluation methods (Table 1). A statistically significant difference was observed in DISCERN scores across question types (general information, symptoms and diagnosis, treatment, and prognosis) (*P*=.01). Treatment and prognosis questions received higher DISCERN ratings compared to general information and symptom/diagnosis questions (*P*<.05). In contrast, no significant differences were detected in evaluator-based ratings (sufficient length, understandability, accuracy, guideline compliance, and patient safety), *JAMA* benchmark scores, or Flesch-Kincaid readability levels (*P*>.05 for all comparisons).

**Table 1.** Distribution of subjective and objective evaluation scores across different clinical question types.

Score type	General information (n=2), mean (SD); median (IQR)	Symptoms and diagnosis (n=6), mean (SD); median (IQR)	Treatment (n=2), mean (SD); median (IQR)	Prognosis (n=5), mean (SD); median (IQR)	Total, mean (SD); median (IQR)	*P* value
Average ratings of raters	22.7 (0.42); 22.7	22.57 (1.3); 23.3	23.3 (0.14); 23.3	22.64 (0.5); 23	22,71 (0.86); 23	.546^a^
DISCERN	23 (8.49); 23	24.83 (6.37); 27.5	31 (0); 30.8	30.8 (0.45); 31	27.40 (5.59); 30	.014^b^
*JAMA* ^c^	0 (0); 0	0 (0); 0	0 (0); 0	0 (0); 0	0 (0); 0	nc^d^
Flesch-Kincaid	17.25 (2.05); 17.25	14.65 (0.96); 17.95	17.95 (3.61); 15.86	15.86 (1.52); 15.4	15.84 (1.94); 15.4	.160^b^

a *P* value obtained from ANOVA test.

b *P* value obtained from the Kruskal-Wallis test.

c *JAMA*: *Journal of the American Medical Association*.

d nc: no computed.

Table 2 presents the interrater agreement and score distribution across key evaluation criteria. The overall ICC across all parameters was 0.772, indicating good consistency among evaluators. When assessed individually, moderate agreement was observed for understandable language (ICC=0.519) and total scores (ICC=0.570), whereas sufficient length (ICC=0.400), accuracy (ICC=0.405), compliance with literature (ICC=0.414), and patient safety (ICC=0.400) demonstrated poor agreement. Statistically significant differences among evaluators were found for sufficient length, accuracy, compliance with literature, and patient safety (all *P*<.001), suggesting subjective variability in scoring across these domains.

**Table 2.** Comparison of evaluator scores across key assessment criteria with interrater agreement values.

Assessment criteria	Enes Karademir, mean^a^ (SD); median (IQR)	Fatih Dolu, mean (SD); median (IQR)	Aydın Hakan Kupeli, mean (SD); median (IQR)	Hüseyin Buyukavci, mean (SD); median (IQR)	Oguzhan Fatih AY, mean (SD); median (IQR)	*P*^b^ value	ICC^c^
Sufficient length	4.33 (0.49); 4.00 (4.00-5.00)	4.53 (0.52); 5.00 (4.00-5.00)	4.8 (0.41); 5.00 (5.00-5.00)	4.2 (0.56); 4.00 (4.00-4.50)	4.87 (0.35); 5.00 (5.00-5.00)	.001^abcdd^	0.400
Understandability	4.67 (0.49); 5.00 (4.00-5.00)	4.6 (0.51); 5.00 (4.00-5.00)	4.93 (0.26); 5.00 (5.00-5.00)	4.93 (0.26); 5.00 (5.00-5.00)	4.87 (0.35); 5.00 (5.00-5.00)	.060	0.519
Accuracy	4.07 (0.59); 4.00 (4.00-4.00)	4.73 (0.46); 5.00 (4.50-5.00)	5 (0); 5.00 (5.00-5.00)	3.87 (0.35); 4.00 (4.00-4.00)	4.87 (0.52); 5.00 (5.00-5.00)	<.001^abcd^	0.405
Compliance with literature	3.07 (0.59); 3.00 (3.00-3.00)	4.47 (0.52); 4.00 (4.00-5.00)	4.87 (0.35); 5.00 (5.00-5.00)	3.87 (0.35); 4.00 (4.00-4.00)	4.87 (0.52); 5.00 (5.00-5.00)	<.001^abc^	0.414
Patient safety	4.73 (0.46); 5.00 (4.50-5.00)	4.67 (0.49); 5.00 (4.00-5.00)	4.93 (0.26); 5.00 (5.00-5.00)	3.93 (0.26); 4.00 (4.00-4.00)	4.87 (0.35); 5.00 (5.00-5.00)	.001^abcd^	0.400
Total	20.87 (2); 21.00 (20.50-22.00)	23 (1.13); 23.00 (22.00-24.00)	24.53 (0.64); 25.00 (24.00-25.00)	20.8 (1.15); 21.00 (20.50-21.50)	24.33 (1.91); 25.00 (25.00-25.00)	.001^abcd^	0.570

a Means followed by distinct small letters (a, b, c, and d) in the same line are significantly different ( *P* <.05).

b *P* value obtained from the Kruskal-Wallis test.

c ICC: intra-correlation coefficient.

d Superscript letters (a, b, c, d, and ab) indicate results of pairwise comparisons. Means sharing the same letter are not significantly different (*P*<.05). For example, “ab” denotes no significant difference from both “a” and “b” groups.

Table 3 shows the evaluator-based scoring patterns across different clinical categories. The overall agreement was poor (ICC=0.199) when clinical categories were not considered separately. However, moderate agreement was found for general information (ICC=0.537) and prognosis-related questions (ICC=0.648), while symptom-based questions demonstrated good agreement (ICC=0.828). Treatment-related questions exhibited moderate consistency (ICC=0.654). These findings suggest that evaluator agreement was strongest for symptom-related questions and weakest for general information questions. Statistically significant differences between evaluators were observed across all clinical categories (*P*<.05).

**Table 3.** Analysis of rater agreement and score variations across different clinical question types.

Scope	Enes Karademir, mean^a^ (SD); median (IQR)	Fatih Dolu, mean (SD); median (IQR)	Hakan Kupeli, mean (SD); median (IQR)	Hüseyin Buyukavci, mean (SD); median (IQR)	Oguzhan Ay, mean (SD); median (IQR)	Total, mean (SD); median (IQR)	*P^b^* value	ICC^c^
General information	21.5 (0.71); 21.5 (21-21)	22.5 (0.71); 22.5 (22-22)	24 (1.41); 24 (23-24)	20.5 (0.71); 20.5 (20-20)	25 (0); 25 (25-25)	22.7 (1.83); 22.5	.015^abcd^	0.537
Prognosis	21.4 (1.14); 21 (21-22)	22.4 (0.89); 22 (22-22)	24.8 (0.45); 25 (25-25)	20.2 (1.64); 21 (19-21)	24.4 (1.34); 25 (25-25)	22.64 (2.08); 22	<.001^abc^	0.648
Symptoms	19.83 (2.71); 20 (17-21)	23.5 (1.38); 24 (23-24)	24.5 (0.55); 24.5 (24-24)	21.17 (0.75); 21 (21-21)	23.83 (2.86); 25 (25-25)	22.57 (2.53); 24	.001^ab^	0.828
Treatment	22 (1.41); 22 (21-22)	23.5 (0.71); 23.5 (23-23)	24.5 (0.71); 24.5 (24-24)	21.5 (0.71); 21.5 (21-21)	25 (0); 25 (25-25)	23.3 (1.57); 23.5	.031^ab^	0.654

a Means followed by distinct small letters (a, b, c, and d) in the same line are significantly different (*P* < .05).

b *P* value obtained from Kruskal-Wallis test.

c ICC: intraclass correlation coefficient.

d Means followed by different superscript letters (a, b, and c) in the same row are significantly different (*P*<.05). The notation "ab" indicates no significant difference from both "a" and "b" groups; "bc" indicates no significant difference from both "b" and "c"; and "abc" indicates no significant difference from groups "a," "b," and "c."

One of the key hypotheses of this study was that the use of highly understandable language might be inversely related to patient safety and adherence to clinical guidelines. Correlation analyses were performed to investigate these associations. Table 4 summarizes the correlations between evaluator ratings for understandability, literature compliance, and patient safety across individual questions, reflecting subjective expert assessments. Table 5 presents the correlation between these evaluator-based parameters and objective assessment tools (DISCERN scores and Flesch-Kincaid readability scores). While no statistically significant overall correlations were found, a strong positive correlation between literature compliance and patient safety was observed for question 8 (*r*=0.968 and *P*<.001), suggesting that in certain contexts, enhanced clinical accuracy may directly benefit patient safety.

**Table 4.** Correlation between evaluator-rated parameters: understandability, literature compliance, and patient safety across questions.

Question	Understandability mean (SD); median^a^ (IQR)	Literature compliance, mean (SD); median (IQR)	Patient safety, mean (SD); median (IQR)	Literature compliance and understandability, r^b^	Patient safety and understandability, r	Literature compliance and patient safety, r	^c^*P^1^* value	*P^2^* value
1	5 (0); 5.0 (5.0-5.0)	4 (0.71); 4.0 (4.0-4.0)	4.4 (0.55); 4.0 (4.0-5.0)	NC	NC	NC	.124	.075
2	4.8 (0.45); 5.0 (5.0-5.0)	4.2 (0.84); 4.0 (4.0-5.0)	4.8 (0.45 5.0 (5.0-5.0)	0.186	−0.250	0.186	.347	—
3	5 (0); 5 (5.0-5.0)	4.4 (0.89); 5 (4.0-5.0)	4.8 (0.45); 5 (5.0-5.0)	NC	NC	0.395	.266	—
4	4.4 (0.55); 4 (4.0-5.0)	3.6 (1.14); 4 (3.0-4.0)	4.4 (0.55); 4 (4.0-5.0)	0.740	0.167	0.152	.107	.0952
5	5 (0); 5 (5.0-5.0)	4.6 (0.55); 5 (4.0-5.0)	4.6 (0.55); 5 4.0-5.0	NC	NC	0.167	.406	—
6	4.8 (0.45); 5 (5.0-5.0)	4.2 (0.84); 4 (4.0-5.0)	4.6 (0.55); 5 (4.0-5.0)	0.745	0.612	0.761	.073	.087
7	5 (0); 5 (5.0-5.0)	4.4 (0.89); 5.0 (4.0-5.0)	4.8 (0.45); 5.0 (5.0-5.0)	NC	NC	0.395	.072	—
8	4.4 (0.55); 4 (4.0-5.0)	4.2 (1.3); 5 (4.0-5.0)	4.6 (0.55); 5 (4.0-5.0)	0.645	0.667	0.968	.406	—
9	4.8 (0.45); 5 (5.0-5.0)	4.6 (0.55); 5 (4.0-5.0)	4.8 (0.45); 5 (5.0-5.0)	0.612	−0.250	0.610	.788	—
10	4.8 (0.45); 5 (5.0-5.0)	4.4 (0.55); 4 (4.0-5.0)	4.8 (0.45); 5 (5.0-5.0)	−0.612	−0.250	0.408	.592	—
11	4.8 (0.45); 5 (5.0-5.0)	4(1); 4 (3.0-5.0)	4.6 (0.89); 5 (5.0-5.0)	0.001	−0.250	0.559	.364	—
12	5 (0); 5 (5.0-5.0)	4.2 (0.84); 4.0 (4.0-5.0)	4.6 (0.55); 5 (4.0-5.0)	NC	NC	0.304	.061	—
13	4.8 (0.45); 5 (5.0-5.0)	4.2 (1.1); 5 (3.0-5.0)	4.4 (0.55); 4 (4.0-5.0)	−0.408	0.400	0.667	.698	—
14	5 (0); 5 (5.0-5.0)	4 (0.71); 4 (4.0-4.0)	4.8 (0.45); 5 (5.0-5.0)	NC	NC	0.100	.027	—
15	4.4 (0.55); 4 (4.0-5.0)	4.4 (0.89); 5 (4.0-5.0)	4.4 (0.55); 5 (4.0-5.0)	0.001	0.167	−0.323	.925	—

a Means followed by distinct small letters (a,b,c, and d) in the same line are significantly different (*P*<.05).

b *r:* Spearman correlation coefficient.

c *P1* value obtained from the Friedman test.

d *P*2 value obtained from the Kruskal-Wallis test.

e NC: non-calculated.

**Table 5.** Correlation analysis between DISCERN and Flesch-Kincaid readability scores.

Correlation statistic		DISCERN score	Flesch-Kincaid score
Mean (SD)		27.40 (SD 5.59)	15.84 (SD 1.94)
r^a^		−0.190	−0.127
*P* value^b^		.498	.653
n^c^		15	15
Correlation (r) with DISCERN		NC^d^	0.057
*P* value		NC	.840
n		NC	15

a r: Spearman correlation coefficient.

b *P* values obtained from the Friedman or Kruskal-Wallis tests, as appropriate.

c n*:* number of observations.

d NC: not calculated.

## Discussion

### Principal Findings

This study assessed the competence of ChatGPT in relation to puerperal mastitis. In our evaluation, all evaluators classified the responses provided across the scope of questions as “excellent.” The DISCERN scoring system indicated that ChatGPT performs better when answering questions related to treatment and prognosis. The Flesch-Kincaid classification indicated that the responses from ChatGPT were comprehensible at the university graduate level (Table 1).

Statistically significant differences were observed in the scores assigned by evaluators across various criteria—including sufficient length (*P*=.001), accuracy (*P*<.001), adherence to the literature (*P*<.001), and patient safety (*P*<.001)—as well as across different question types, such as general information (*P*=.015), prognosis (*P*<.001), symptoms (*P*=.001), and treatment (*P*=.031). These findings suggest that the evaluation of ChatGPT responses was influenced by the evaluators’ individual perspectives, especially for broader and more context-dependent inquiries. The relatively smaller difference among raters in the criterion of understandable language (*P*=.060) suggests that this parameter may be more amenable to objective assessment. Taken together, the inter-rater variability highlights an inherent limitation of subjective evaluation frameworks and underscores the need for the development of more standardized and objective rating tools for assessing AI-generated health content. Establishing validated scoring protocols may improve consistency and reliability in future assessments involving human raters.

### Comparison With Prior Work

The literature contains numerous evaluations of ChatGPT’s accuracy in response to inquiries about both malignant and benign diseases. AI-generated data has shown precise detection of osteoarthritis, though technical support is still necessary for some specific questions [14]. Data regarding urothelial carcinoma from AI are currently limited, with reliable information mainly focused on the epidemiology and risk factors [15]. In breast cancer oncology treatment, AI offers restricted insights into particular clinical scenarios and postoperative conditions [16]. In the pediatric field, Wei noted that while ChatGPT’s performance was adequate for general inquiries, it was insufficient in specific clinical contexts [17]. Our study represents the first assessment of ChatGPT’s responses regarding puerperal mastitis, with no existing research available for comparison.

In relation to another one of our hypotheses, in our analysis of the correlation among understandable language, patient safety, and compliance with the literature, we observed a positive correlation between the parameters of compliance and patient safety related to the 8th question, which is focused on symptoms and diagnosis (Table 4). The inability to statistically verify one of our hypotheses may stem from the limited number of questions (n=15) and the subjective nature of the raters’ evaluations. Nonetheless, no statistically significant relationship was identified between the DISCERN score, a relatively objective indicator of technical proficiency, and the Flesch-Kincaid Reading Difficulty score, which assesses comprehensibility, for each question (Table 5). However, Al-Ashwal et al [18] indicated that using simple language may result in the omission of critical information regarding disease. Various indexes, such as the Flesch-Kincaid Index, Simple Measure of Gobbledygook index [19], and Gunning Fog Index [17], have been used in the literature to assess readability and understandability. Valencia [20] stated that the AI response might be effective for the Flesch-Kincaid index in the context of kidney donation information and studies related to ChatGPT. He noted that this was a quantitative evaluation and may not encompass all nuances; thus, it may lack generalizability. It is noted that unclear online health information negatively affects patient adherence to treatment [8,21]. We believe that it is essential for the information supplied by ChatGPT to be both precise and comprehensible to patients. At present, it has been argued that ChatGPT has the potential to enhance patient outcomes and health literacy [22]. In our study, we used the Flesch-Kincaid Index and determined that the responses provided by ChatGPT were comprehensible at the university level. While this suggests a high level of linguistic and grammatical complexity, it may limit accessibility for patients with lower health literacy. Although the Flesch-Kincaid Index has its limitations—particularly in evaluating medical jargon and sentence structure—it remains one of the most widely accepted readability tools in digital health research. Previous studies evaluating AI-generated content have reported similar findings [8,17,23][]. We believe that improving the readability of ChatGPT responses, particularly by simplifying language without compromising medical accuracy, is essential for optimizing patient education and safety.

One of our central hypotheses was that ChatGPT could contribute meaningfully to clinical decision-making and patient management in outpatient settings. Beyond its role in health education and patient information, recent studies have highlighted ChatGPT’s emerging potential in various clinical domains. In abdominopelvic surgery, it has been proposed as a tool for enhancing patient-physician communication, preoperative counseling, and postoperative care guidance [24]. Similarly, in gastroenterology, its capacity for triage support, education on endoscopic procedures, and initial interpretation of laboratory findings has been explored, though concerns remain regarding accuracy and source citation [25]. The cardiology field has also recognized its utility for drafting discharge summaries and answering patient queries, particularly in heart failure management and preventive care [26]. Furthermore, in a comparative study within evidence-based dentistry, ChatGPT-4 demonstrated superior performance over other LLMs, particularly in alignment with clinical guidelines, suggesting its value as a supportive assistant in routine decision-making [27]. These findings collectively underline that while ChatGPT is not a replacement for clinical judgment, its structured integration into practice—especially for low-risk guidance and standardized educational content—may enhance efficiency, patient comprehension, and adherence to evidence-based care models. One of the hypotheses of this study was that ChatGPT could contribute meaningfully to clinical decision-making and patient management. However, the integration of AI into health care practices raises several ethical concerns that warrant careful consideration. First, the uncertainty regarding liability in the event of harm resulting from AI-generated recommendations remains unresolved. It is unclear whether responsibility would lie with the clinician, the AI developer, or the institution using the system. This issue has been widely discussed across different fields of medicine, emphasizing the necessity for clear accountability structures [28]. Second, transparency in AI decision-making is a fundamental requirement for clinical trust [28,29]. It is recommended that AI systems not only disclose their involvement in clinical processes but also provide a traceable rationale for their outputs. This approach may help reduce ambiguity related to AI-derived recommendations and enhance professional confidence in their use. Third, algorithmic bias—arising from limitations or imbalances in the datasets used to train AI—can lead to inequitable outcomes for certain patient populations. Although this dimension was not directly evaluated in our study, it is a well-recognized risk and must be considered in future applications of AI in medicine [28,29]. Moreover, long-term dependence on AI may lead to the phenomenon of “automation bias,” in which clinicians overly rely on AI recommendations, potentially diminishing their own diagnostic acumen and clinical judgment [30]. This may affect future training and critical thinking in medical education, especially if AI systems are perceived as replacements rather than adjuncts to human expertise.

These considerations underline the need for specialty-specific ethical guidelines that address transparency, accountability, bias mitigation, and appropriate use of AI in clinical practice. While our study found that ChatGPT may provide accurate and comprehensible responses in the context of puerperal mastitis, further ethical and regulatory frameworks are essential to ensure its safe and equitable integration into health care delivery.

### Limitations

This study has several notable limitations. First, each question was submitted to ChatGPT only once, and the initial response was evaluated without any iterative prompting or follow-up queries. In real-world clinical settings, both health care professionals and patients often engage in multiturn interactions to clarify ambiguities, which may lead to more comprehensive or accurate outputs. Our single-response approach, while practical for standardization, may not fully capture the potential of conversational AI tools in dynamic outpatient scenarios. Second, the assessment was conducted on a specific version of ChatGPT (GPT-4o mini) at a single time point using the publicly accessible platform. Since LLMs are frequently updated and refined, the findings of this study may not be generalizable to future versions or other AI models. In addition, because the chatbot does not cite references or provide source transparency, its outputs may lack traceability—particularly relevant when considering adherence to clinical guidelines and patient safety. Third, although the evaluation was conducted by a team of general surgeons and surgical residents, the rating process remains subject to individual clinical judgment. Parameters such as “accuracy” or “patient safety”—despite being guided by current literature—may still reflect inherent evaluator bias. Although inter-rater reliability was statistically analyzed, the subjective nature of certain domains introduces variability and limits reproducibility. Finally, the number of questions (n=15) was based on prior literature and feasibility considerations; however, it limits the breadth of topics and clinical diversity that could be assessed. Broader question sets and diverse clinical domains would be valuable in future studies to enhance generalizability.

### Conclusion

Our findings indicate that ChatGPT has the potential to serve as an effective assistant in general surgery outpatient settings by providing responses that are not only comprehensible for patients but also largely aligned with current clinical literature. This dual competency—offering medically relevant and literature-consistent information while maintaining clarity—positions ChatGPT as a promising tool in supporting clinical decision-making and enhancing the efficiency of patient management.

To fully realize this potential, future research should focus on improving the consistency and reliability of AI outputs across diverse clinical topics. Further studies should also explore strategies for ensuring up-to-date medical knowledge within language models and evaluate their integration into real-time clinical workflows. These efforts are essential for safely expanding the role of AI in evidence-based, patient-centered care.

We assessed ChatGPT’s performance in addressing questions related to puerperal mastitis, a condition that represents a substantial portion of the workload in general surgery outpatient clinics, in the context of the prospective role of AI in the health care system. Our findings indicate that ChatGPT provides adequate information regarding puerperal mastitis. While the parameters used to assess for understandability have inherent limitations, it is essential to use more comprehensible language to achieve favorable outcomes that may be broadly disseminated within society. There is a need for more comprehensive, technical, and methodologically sound studies to integrate AI into health care systems.

## Supplementary material

10.2196/68980 Multimedia Appendix 1 Turkish and English versions of the questions used in the study. This appendix presents the original Turkish questions submitted to ChatGPT and their English translations, categorized by clinical content.

10.2196/68980 Multimedia Appendix 2 Original Turkish responses generated by ChatGPT. This appendix presents the original, unedited responses generated by ChatGPT (GPT-40 mini) for each of the 15 Turkish-language questions used in the study. These responses were obtained without any postprocessing or follow-up prompts and reflect the artificial intelligence’s standalone performance in providing patient-oriented information.

10.2196/68980 Multimedia Appendix 3 Evaluation table containing assessor scores and summary metrics. This appendix includes the scoring data provided by each of the five evaluators, including categorical breakdowns for sufficient length, understandability, accuracy, adherence to guidelines, and patient safety.

10.2196/68980 Multimedia Appendix 4 Aggregated evaluator scores by question and comparative analysis. This appendix presents mean scores for each question across the evaluation domains and includes comparative statistical analyses among question categories.

## Abbreviations

AI: artificial intelligence
ICC: intraclass correlation coefficient
*JAMA*: *Journal of the American Medical Association*
LLM: large language model
WHO: World Health Organization

## References

[1] Amir LH, Crawford SB, Cullinane M, Grzeskowiak LE (2024). General practitioners’ management of mastitis in breastfeeding women: a mixed method study in Australia. BMC Prim Care.

[2] Lin YC, Lee YL, Chen YH, Tsao SM, Wang WY (2024). Puerperal mastitis caused by limited community-associated methicillin-resistant *Staphylococcus aureus* (CA-MRSA) clones. Front Med (Lausanne).

[3] Wilson E, Woodd SL, Benova L (2020). Incidence of and risk factors for lactational mastitis: a systematic review. J Hum Lact.

[4] Secinaro S, Calandra D, Secinaro A, Muthurangu V, Biancone P (2021). The role of artificial intelligence in healthcare: a structured literature review. BMC Med Inform Decis Mak.

[5] Li X, Tian D, Li W (2021). Artificial intelligence-assisted reduction in patients’ waiting time for outpatient process: a retrospective cohort study. BMC Health Serv Res.

[6] Liu J, Wang C, Liu S (2023). Utility of ChatGPT in clinical practice. J Med Internet Res.

[7] Potapenko I, Boberg-Ans LC, Stormly Hansen M, Klefter ON, van Dijk EHC, Subhi Y (2023). Artificial intelligence-based chatbot patient information on common retinal diseases using ChatGPT. Acta Ophthalmol.

[8] Yeo YH, Samaan JS, Ng WH (2023). Assessing the performance of ChatGPT in answering questions regarding cirrhosis and hepatocellular carcinoma. Clin Mol Hepatol.

[9] Scheetz J, Rothschild P, McGuinness M (2021). A survey of clinicians on the use of artificial intelligence in ophthalmology, dermatology, radiology and radiation oncology. Sci Rep.

[10] Charnock D, Shepperd S, Needham G, Gann R (1999). DISCERN: an instrument for judging the quality of written consumer health information on treatment choices. J Epidemiol Community Health.

[11] Silberg WM, Lundberg GD, Musacchio RA (1997). Assessing, controlling, and assuring the quality of medical information on the internet: caveant lector et viewor--let the reader and viewer beware. JAMA.

[12] Gill PS (2013). Prescription painkillers and controlled substances: an appraisal of drug information provided by six US pharmacies. Drug Healthc Patient Saf.

[13] Koo TK, Li MY (2016). A guideline of selecting and reporting intraclass correlation coefficients for reliability research. J Chiropr Med.

[14] Mika AP, Martin JR, Engstrom SM, Polkowski GG, Wilson JM (2023). Assessing ChatGPT responses to common patient questions regarding total hip arthroplasty. J Bone Joint Surg Am.

[15] Łaszkiewicz J, Krajewski W, Tomczak W (2024). Performance of ChatGPT in providing patient information about upper tract urothelial carcinoma. Contemp Oncol (Pozn).

[16] Stalp JL, Denecke A, Jentschke M, Hillemanns P, Klapdor R (2024). Quality of ChatGPT-generated therapy recommendations for breast cancer treatment in gynecology. Curr Oncol.

[17] Wei Q, Wang Y, Yao Z (2023). Evaluation of ChatGPT’s performance in providing treatment recommendations for pediatric diseases. Pediatric Discovery.

[18] Al-Ashwal FY, Zawiah M, Gharaibeh L, Abu-Farha R, Bitar AN (2023). Evaluating the sensitivity, specificity, and accuracy of ChatGPT-3.5, ChatGPT-4, Bing AI, and Bard against conventional drug-drug interactions clinical tools. Drug Healthc Patient Saf.

[19] Miao J, Thongprayoon C, Cheungpasitporn W (2023). Assessing the accuracy of ChatGPT on core questions in glomerular disease. Kidney Int Rep.

[20] Garcia Valencia OA, Thongprayoon C, Miao J (2024). Empowering inclusivity: improving readability of living kidney donation information with ChatGPT. Front Digit Health.

[21] Tan JY, Tan YC, Yap D (2023). Readability and quality of online patient health information on parotidectomy. J Laryngol Otol.

[22] Sallam M (2023). ChatGPT utility in healthcare education, research, and practice: systematic review on the promising perspectives and valid concerns. Healthcare (Basel).

[23] Subramanian T, Araghi K, Amen TB (2024). Chat generative pretraining transformer answers patient-focused questions in cervical spine surgery. Clin Spine Surg.

[24] Goglia M, Pace M, Yusef M (2024). Artificial intelligence and ChatGPT in abdominopelvic surgery: a systematic review of applications and impact. In Vivo.

[25] Klang E, Sourosh A, Nadkarni GN, Sharif K, Lahat A (2023). Evaluating the role of ChatGPT in gastroenterology: a comprehensive systematic review of applications, benefits, and limitations. Therap Adv Gastroenterol.

[26] Sharma A, Medapalli T, Alexandrou M, Brilakis E, Prasad A (2024). Exploring the role of ChatGPT in cardiology: a systematic review of the current literature. Cureus.

[27] Giannakopoulos K, Kavadella A, Aaqel Salim A, Stamatopoulos V, Kaklamanos EG (2023). Evaluation of the performance of generative AI large language models ChatGPT, Google Bard, and Microsoft Bing chat in supporting evidence-based dentistry: comparative mixed methods study. J Med Internet Res.

[28] Wang C, Liu S, Yang H, Guo J, Wu Y, Liu J (2023). Ethical considerations of using ChatGPT in health care. J Med Internet Res.

[29] Kenig N, Monton Echeverria J, Rubi C (2024). Ethics for AI in plastic surgery: guidelines and review. Aesthetic Plast Surg.

[30] Abdullah YI, Schuman JS, Shabsigh R, Caplan A, Al-Aswad LA (2021). Ethics of artificial intelligence in medicine and ophthalmology. Asia Pac J Ophthalmol (Phila).

