# Supplementary material for: Evaluation of ChatGPT-4 as an Online Outpatient Assistant in Puerperal Mastitis Management: Content Analysis of an Observational Study
Source: JMIR Med Inform. 2025 Jul 24;13:e68980. doi: 10.2196/68980 (PMC12288767; doi:10.2196/68980)
Supplement: Multimedia Appendix 1 [file medinform-v13-e68980-s001.docx]

Table 1 : Asked questions and their English translations

| **Question number** | **Kapsam** | **Soru** | **Context** | **English translation of question** |
| --- | --- | --- | --- | --- |
| 1 | Genel Bilgi | Puerperal mastit nedir? | General Information | What is puerperal mastitis? |
| 2 | Genel Bilgi | Puerperal mastitin yaygın nedenleri nelerdir? | General Information | What are the common causes of puerperal mastitis? |
| 3 | Semptom ve Teşhis | Puerperal mastitin belirtileri nelerdir? | Symptoms and Diagnosis | What are the symptoms of puerperal mastitis? |
| 4 | Semptom ve Teşhis | Memede mastit olması sütün kesilmesine neden olur mu? | Symptoms and Diagnosis | Does mastitis in the breast cause milk supply to stop? |
| 5 | Semptom ve Teşhis | Puerperal mastit nasıl teşhis edilir? | Symptoms and Diagnosis | How is puerperal mastitis diagnosed? |
| 6 | Semptom ve Teşhis | Puerperal mastit ile meme apsesi arasındaki fark nedir? | Symptoms and Diagnosis | What is the difference between puerperal mastitis and breast abscess? |
| 7 | Semptom ve Teşhis | Puerperal mastit ve süt birikimi (stazı) arasındaki fark nedir? | Symptoms and Diagnosis | What is the difference between puerperal mastitis and milk stasis? |
| 8 | Semptom ve Teşhis | Granülomatöz mastit puerperal mastit ile karışabilir mi? | Symptoms and Diagnosis | Can granulomatous mastitis be confused with puerperal mastitis? |
| 9 | Tedavi | Puerperal mastit nasıl tedavi edilir? | Treatment | How is puerperal mastitis treated? |
| 10 | Tedavi | Antibiyotik tedavisi puerperal mastit için her zaman gerekli midir? | Treatment | Is antibiotic treatment always necessary for puerperal mastitis? |
| 11 | Prognoz | Puerperal mastit olan bir anne emzirmeye devam edebilir mi? | Prognosis | Can a mother with puerperal mastitis continue breastfeeding? |
| 12 | Prognoz | Puerperal mastitin tekrarlama riski var mı? | Prognosis | What is the risk of recurrence for puerperal mastitis? |
| 13 | Prognoz | Puerperal mastit uzun vadede hangi komplikasyonlara yol açabilir? | Prognosis | What long-term complications can puerperal mastitis cause? |
| 14 | Prognoz | Mastit tedavisinden sonra memede sertlik kalır mı? | Prognosis | Does mastitis leave hardness in the breast after treatment? |
| 15 | Prognoz | Mastit oluşumunu önlemek için neler yapılmalıdır? | Prognosis | What can be done to prevent mastitis? |
