# Supplementary material for: Evaluation of ChatGPT-4 as an Online Outpatient Assistant in Puerperal Mastitis Management: Content Analysis of an Observational Study
Source: JMIR Med Inform. 2025 Jul 24;13:e68980. doi: 10.2196/68980 (PMC12288767; doi:10.2196/68980)
Supplement: Multimedia Appendix 2 [file medinform-v13-e68980-s002.docx]

| Soru No |
| --- |
| 1) Puerperal mastit nedir? |
| 2) Puerperal mastitin yaygın nedenleri nelerdir? |
| 3) Puerperal mastitin belirtileri nelerdir? |
| 4)Memede mastit olması sütün kesilmesine neden olur mu? |
| 5) Puerperal mastit nasıl teşhis edilir? |
| 6) Puerperal mastit ile meme apsesi arasındaki fark nedir? |
| 7) Puerperal mastit ve süt birikimi (stazı) arasındaki fark nedir? |
| 8) Granülomatöz mastit puerperal mastit ile karışabilir mi? |
| 9) Puerperal mastit nasıl tedavi edilir? |
| 10) Antibiyotik tedavisi puerperal mastit için her zaman gerekli midir? |
| 11) Puerperal mastit olan bir anne emzirmeye devam edebilir mi? |
| 12) Puerperal mastitin tekrarlama riski var mı? |
| 13) Puerperal mastit uzun vadede hangi komplikasyonlara yol açabilir? |
| 14)Mastit tedavisinden sonra memede sertlik kalır mı? |
| 15)Mastit oluşumunu önlemek için neler yapılmalıdır? |

1) Puerperal mastit nedir?

**ChatGPT said:**

ChatGPT

Puerperal mastit, doğumdan sonraki dönemde görülen bir meme iltihabıdır. Genellikle doğum sonrası ilk birkaç hafta içinde ortaya çıkar ve genellikle emzirme sırasında meme ucu çatlakları veya bakteriyel enfeksiyonlar nedeniyle oluşur. Belirtileri arasında meme üzerinde ağrı, şişlik, kızarıklık ve bazen ateş bulunur. Tedavi edilmezse, mastit ciddi komplikasyonlara yol açabilir, bu yüzden erken müdahale önemlidir. Tedavi genellikle antibiyotikler, ağrı kesiciler ve meme sağlığını destekleyici yöntemleri içerir.

**You said:**

2) Puerperal mastitin yaygın nedenleri nelerdir?

**ChatGPT said:**

ChatGPT

Puerperal mastitin yaygın nedenleri şunlardır:

1. **Meme Ucu Çatlakları veya Yaralar:** Emzirme sırasında meme ucu çatlakları veya yaralar, bakterilerin (genellikle Staphylococcus aureus) meme dokusuna girmesine ve enfeksiyon oluşturmasına neden olabilir.
2. **Süt Birikimi (Laktasyonel Staz):** Meme içinde süt birikmesi, süt kanallarında tıkanmalara ve enfeksiyonlara yol açabilir. Bu, süt akışının engellenmesine ve iltihaplanmaya neden olabilir.
3. **Bakteriyel Enfeksiyonlar:** Mastit genellikle ciltte bulunan bakterilerin (özellikle Staphylococcus aureus ve Streptococcus türleri) meme dokusuna geçmesi sonucu ortaya çıkar.
4. **Yetersiz Emzirme veya Sıkışmış Süt:** Emzirmenin düzenli olmaması veya bebeğin memeyi yeterince etkili şekilde emmemesi süt akışını engelleyebilir ve bu durum iltihaplanmaya yol açabilir.
5. **Meme Temizliğine Dikkat Edilmemesi:** Meme temizliğine özen gösterilmemesi, bakteriyel enfeksiyon riskini artırabilir.
6. **Hormonel Değişiklikler:** Doğum sonrası hormonel değişiklikler de meme dokusunda hassasiyete ve enfeksiyonlara yatkınlığa neden olabilir.

Bu nedenler genellikle birbirini etkileyebilir ve mastit gelişimine zemin hazırlayabilir. Erken teşhis ve uygun tedavi, komplikasyonları önlemeye yardımcı olabilir.

**You said:**

3) Puerperal mastitin belirtileri nelerdir?

**ChatGPT said:**

ChatGPT

Puerperal mastitin belirtileri genellikle aşağıdaki gibidir:

1. **Meme Üzerinde Ağrı ve Hassasiyet:** Enfekte olan memede ağrı, rahatsızlık ve hassasiyet hissedilebilir. Bu ağrı, genellikle lokalize olup, şiddeti zamanla artabilir.
2. **Meme Şişliği ve Kızarıklık:** Enfekte bölgedeki meme dokusunda şişlik, kızarıklık ve ısı artışı görülebilir. Bu bölgede sıcaklık hissi de olabilir.
3. **Ateş ve Titreme:** Mastit genellikle sistemik belirtilerle birlikte gelir, bu nedenle ateş, titreme ve genel bir halsizlik olabilir.
4. **Genel Yorgunluk ve Halsizlik:** Enfeksiyon, genel bir yorgunluk ve halsizlik hissine neden olabilir.
5. **Süt Kanalında Sertlik:** Meme içinde sert, düzensiz bölgeler veya süt kanallarında tıkanıklık hissi olabilir. Bu, genellikle enfekte olan bölgede daha belirgin olabilir.
6. **Meme Ucu Akıntısı:** Enfekte memeden koyu renkli veya pürülan (iltihaplı) bir akıntı olabilir. Bu, iltihabın şiddetine bağlı olarak değişebilir.
7. **Bebekte Emzirme Zorluğu:** Emzirme sırasında bebeğin memeyi etkili bir şekilde emmemesi veya emzirmenin ağrılı olması mastitin bir belirtisi olabilir.

Bu belirtiler ortaya çıktığında, erken teşhis ve tedavi için bir sağlık profesyoneline başvurmak önemlidir. Tedavi edilmediğinde mastit ciddi komplikasyonlara yol açabilir, bu yüzden belirtiler fark edildiğinde hızlıca hareket edilmelidir.

**You said:**

4)Memede mastit olması sütün kesilmesine neden olur mu?

**ChatGPT said:**

ChatGPT

Evet, memede mastit olması sütün kesilmesine neden olabilir. Mastit, süt üretimini ve akışını etkileyebilir ve bazı durumlarda sütün tamamen kesilmesine yol açabilir. İşte mastitin süt üretimi üzerindeki etkileri:

1. **Süt Akışında Düşüş:** Mastit, süt kanallarında tıkanıklıklara ve iltihaplanmalara yol açarak süt akışını zorlaştırabilir. Bu durum, sütün memeden akışını engelleyebilir ve süt üretimini etkileyebilir.
2. **Süt Üretiminde Azalma:** Enfeksiyon ve iltihaplanma, süt üretimini etkileyebilir. Mastit sırasında vücudun enfeksiyonla savaşması, süt üretiminde azalmaya neden olabilir.
3. **Emzirme Zorluğu:** Mastit, emzirme sırasında ağrıya ve rahatsızlığa neden olabilir. Bu durum, emzirmenin zorlaşmasına ve dolayısıyla süt üretiminin azalmasına yol açabilir.
4. **Meme Sağlığı Sorunları:** Meme dokusundaki iltihap ve enfeksiyon, meme sağlığını etkileyebilir ve süt üretimini olumsuz yönde etkileyebilir.

Tedavi edilmediğinde mastit ciddi komplikasyonlara neden olabilir ve süt üretimi üzerinde kalıcı etkiler bırakabilir. Ancak uygun tedavi ile çoğu zaman süt üretimi yeniden normale dönebilir. Mastitin tedavisi genellikle antibiyotikler, ağrı kesiciler ve uygun emzirme teknikleri içerir. Erken müdahale ve doğru tedavi ile süt üretimi genellikle tekrar eski düzeyine dönebilir.

**You said:**

5) Puerperal mastit nasıl teşhis edilir?

**ChatGPT said:**

ChatGPT

Puerperal mastit teşhisi genellikle klinik değerlendirme ve bazı ek testlerle yapılır. İşte teşhis sürecinde izlenen adımlar:

1. **Tıbbi Geçmiş ve Semptomlar:** Doktor, hastanın tıbbi geçmişini ve mevcut semptomlarını değerlendirir. Mastit belirtileri arasında meme üzerindeki ağrı, şişlik, kızarıklık, ateş ve genel halsizlik gibi belirtiler olup olmadığına bakılır.
2. **Fizik Muayene:** Doktor, meme dokusunu fiziksel olarak muayene eder. Bu muayenede şişlik, kızarıklık, sıcaklık artışı ve ağrı gibi belirtiler aranır. Ayrıca, meme üzerinde sertleşmiş bölgeler veya abse oluşumları kontrol edilir.
3. **Meme Ultrasonu:** Meme ultrasonu, iltihaplanma alanlarını ve süt kanallarındaki tıkanıklıkları veya abse oluşumlarını değerlendirmek için kullanılır. Bu test, mastitin tanısını doğrulamada yardımcı olabilir ve cerrahi müdahale gerekip gerekmediğini belirlemede de faydalı olabilir.
4. **Kan Testleri:** Kan testleri, enfeksiyonun varlığını ve şiddetini değerlendirmek için yapılır. Beyaz kan hücresi sayısı (WBC) ve diğer iltihap belirteçleri (örneğin, C-reaktif protein) yüksek olabilir.
5. **Süt Kültürü:** Eğer meme akıntısı varsa, bu akıntıdan örnek alınarak laboratuvar testleri yapılabilir. Süt kültürü, enfeksiyona neden olan bakteriyel patojenleri belirlemeye yardımcı olabilir ve uygun antibiyotik tedavisinin seçilmesinde rehberlik eder.

Bu testler ve değerlendirmeler, doktorun doğru bir teşhis koymasına ve uygun tedavi yöntemlerini belirlemesine yardımcı olur. Mastitin erken teşhisi ve tedavisi, komplikasyonları önlemeye ve iyileşme sürecini hızlandırmaya yardımcı olabilir.

**You said:**

6) Puerperal mastit ile meme apsesi arasındaki fark nedir?

**ChatGPT said:**

ChatGPT

Puerperal mastit ve meme apsesi, meme dokusunda enfeksiyon ve iltihaplanma ile ilişkili durumlardır, ancak aralarında önemli farklar vardır. İşte bu iki durum arasındaki temel farklar:

**Puerperal Mastit**

1. **Tanım:** Puerperal mastit, doğum sonrası dönemde görülen meme iltihabıdır. Genellikle emzirme sırasında meydana gelir ve meme dokusundaki iltihaplanmayı ifade eder.
2. **Belirtiler:**
   - Meme üzerinde ağrı, hassasiyet, şişlik ve kızarıklık.
   - Ateş, titreme ve genel halsizlik.
   - Meme akıntısı veya süt akışında değişiklik olabilir.
3. **Nedenler:** Genellikle bakteriyel enfeksiyonlar, meme ucu çatlakları, süt kanalı tıkanıklıkları veya yetersiz emzirme gibi nedenlerle oluşur.
4. **Tedavi:** Antibiyotikler, ağrı kesiciler, sıcak kompresler ve sık sık emzirme veya süt pompalama ile tedavi edilir. Mastitin erken tedavisi genellikle tamamen iyileşme sağlar.

**Meme Apsesi**

1. **Tanım:** Meme apsesi, mastitin ilerlemesi sonucu oluşan, içinde irin bulunan bir abseye verilen isimdir. Mastit tedavi edilmezse veya yeterince etkili bir tedavi yapılmazsa, iltihaplı bölgede irin birikir ve abseye dönüşür.
2. **Belirtiler:**
   - Şiddetli ağrı, özellikle absenin olduğu bölgede.
   - Meme üzerinde belirgin bir şişlik ve kızarıklık.
   - Ateş, titreme ve genel halsizlik.
   - Absenin bulunduğu bölgede sertlik veya kitle hissi.
   - İrinli akıntı veya meme üzerinde kötü kokulu bir akıntı olabilir.
3. **Nedenler:** Genellikle mastitin ilerlemesi sonucu bakteriyel enfeksiyonun daha ciddi bir aşamaya geçmesi ve irin oluşması ile ortaya çıkar.
4. **Tedavi:** Meme apsesinin tedavisi genellikle cerrahi müdahale gerektirir. Absenin drenajı, antibiyotik tedavisi ve ağrı yönetimi uygulanır. Erken tedavi ve uygun drenaj işlemi genellikle iyileşme sürecini hızlandırır.

Özetle, puerperal mastit iltihaplanma ve enfeksiyonun erken aşamasıdır, genellikle medikal tedavi ile iyileşebilir. Meme apsesi, mastitin ilerlemesi ve irin birikimi sonucunda oluşur ve genellikle cerrahi müdahale gerektirir.

**You said:**

7) Puerperal mastit ve süt birikimi (stazı) arasındaki fark nedir?

**ChatGPT said:**

ChatGPT

Puerperal mastit ve süt birikimi (stazı) hem meme ile ilgili sorunlardır, ancak farklı nedenlere ve tedavi yaklaşımlarına sahiptir. İşte bu iki durum arasındaki temel farklar:

**Puerperal Mastit**

1. **Tanım:** Puerperal mastit, doğum sonrası dönemde meydana gelen meme iltihabıdır. Genellikle bakteriyel enfeksiyon nedeniyle oluşur ve iltihaplanma, enfeksiyon ve sistemik belirtiler ile karakterizedir.
2. **Belirtiler:**
   - Meme üzerinde ağrı, hassasiyet, şişlik ve kızarıklık.
   - Ateş, titreme, genel halsizlik.
   - Meme üzerinde sıcaklık artışı ve bazen irinli akıntı.
   - Enfekte olan bölgede sertlik ve genişleyen bir şişlik olabilir.
3. **Nedenler:** Genellikle bakteriyel enfeksiyon (genellikle Staphylococcus aureus) sonucu oluşur. Bakteriler, meme ucu çatlakları, tıkanmış süt kanalları veya diğer giriş yollarından memeye girer.
4. **Tedavi:** Antibiyotikler, ağrı kesiciler, sıcak kompresler ve uygun emzirme veya süt pompalama teknikleri ile tedavi edilir. Ciddi vakalarda, apse oluşumu varsa cerrahi müdahale gerekebilir.

**Süt Birikimi (Stazı)**

1. **Tanım:** Süt birikimi, süt kanallarında süt birikmesi ve akışının engellenmesi ile oluşur. Bu durum genellikle laktasyon sırasında süt akışının düzensizliği veya engellenmesi nedeniyle ortaya çıkar.
2. **Belirtiler:**
   - Meme üzerinde belirgin bir şişlik veya sertlik.
   - Ağrı ve rahatsızlık, genellikle lokalize.
   - Meme dokusunda belirgin bir sertleşme veya düzensizlik hissi.
   - Süt akışında düzensizlik veya azalma olabilir.
3. **Nedenler:** Genellikle süt kanallarında tıkanıklık, yetersiz emzirme, bebeğin memeyi etkili bir şekilde emmemesi veya emzirme sıklığındaki değişiklikler nedeniyle oluşur. Süt birikimi genellikle mastit gelişmeden önce bir sorun olarak ortaya çıkabilir.
4. **Tedavi:** Süt birikimi genellikle emzirme tekniklerinin düzeltilmesi, sık sık emzirme veya süt pompalama, sıcak kompresler ve masaj ile tedavi edilir. Süt birikimi genellikle mastit gelişmeden çözülür ve daha ciddi bir duruma yol açmadan iyileşebilir.

**Özet**

- **Puerperal Mastit:** Bakteriyel enfeksiyon sonucu meydana gelen meme iltihabıdır. Genellikle sistemik belirtilerle birlikte gelir ve tedavi edilmediğinde ciddi komplikasyonlara yol açabilir.
- **Süt Birikimi (Stazı):** Süt kanallarında biriken süt ve süt akışının engellenmesidir. Genellikle emzirme veya süt pompalama yöntemleri ile düzeltilir ve mastit gelişmeden çözülür.

Her iki durumda da meme sağlığını korumak için erken teşhis ve uygun tedavi önemlidir.

**You said:**

8) Granülomatöz mastit puerperal mastit ile karışabilir mi?

**ChatGPT said:**

ChatGPT

Evet, granülomatöz mastit ve puerperal mastit belirtileri bazı benzerlikler taşıdığı için karışabilir. Ancak, bu iki durum farklı patolojik süreçlerle ortaya çıkar ve tedavi yaklaşımları da farklıdır. İşte granülomatöz mastit ve puerperal mastit arasındaki farklar ve bu durumların karışabileceği noktalar:

**Granülomatöz Mastit**

1. **Tanım:** Granülomatöz mastit, nadir görülen ve meme dokusunda granülom (iltihaplı, yabancı cisim reaksiyonu) oluşumuyla karakterize bir durumdur. Genellikle autoimmun veya yabancı cisim reaksiyonları ile ilişkilidir ve bazen ilaçlar (örneğin, kontraseptifler) veya şiddetli enfeksiyonlar sonrasında ortaya çıkabilir.
2. **Belirtiler:**
   - Meme üzerinde ağrı, şişlik ve sertlik.
   - Kızarıklık, bazen iltihaplı bölgelerde çıban veya apse benzeri oluşumlar.
   - İltihaplı bölgelerde düzensiz kitleler veya nodüller.
   - Genellikle ateş ve sistemik semptomlar olmayabilir, ancak yerel iltihap belirtileri belirgin olabilir.
3. **Tanı:** Granülomatöz mastit, genellikle biyopsi ve histopatolojik inceleme ile teşhis edilir. Meme dokusundan alınan örnekler, granülom oluşumlarını ve diğer patolojik değişiklikleri belirlemeye yardımcı olur.
4. **Tedavi:** Tedavi, duruma bağlı olarak değişir ve genellikle steroidler gibi immünsupresif ilaçlar kullanılır. Antibiyotikler genellikle etkili değildir çünkü granülomatöz mastit genellikle enfeksiyon yerine immün yanıtla ilişkilidir.

**Puerperal Mastit**

1. **Tanım:** Puerperal mastit, doğum sonrası dönemde meydana gelen bakteriyel enfeksiyon nedeniyle meme iltihabıdır. Emzirme sırasında meydana gelir ve bakteriyel enfeksiyon sonucu oluşur.
2. **Belirtiler:**
   - Meme üzerinde ağrı, şişlik, kızarıklık ve ısı artışı.
   - Ateş, titreme ve genel halsizlik.
   - Meme üzerinde sertlik ve bazen irinli akıntı olabilir.
3. **Tanı:** Puerperal mastit genellikle klinik değerlendirme ve bazen süt kültürü ile teşhis edilir. İltihap belirtileri ve sistemik semptomlar mastit tanısını destekler.
4. **Tedavi:** Antibiyotikler, sıcak kompresler, ağrı kesiciler ve uygun emzirme teknikleri ile tedavi edilir. Ciddi vakalarda abse oluşumu varsa cerrahi drenaj gerekebilir.

**Karışabilme Durumları:**

- **Benzer Belirtiler:** Her iki durum da meme ağrısı, şişlik ve kızarıklık gibi benzer belirtilerle kendini gösterebilir.
- **Tanı Zorluğu:** Granülomatöz mastit ve puerperal mastit arasındaki ayırt edici farklar genellikle klinik değerlendirme ve laboratuvar testleri ile ortaya çıkar. Granülomatöz mastit histopatolojik inceleme gerektirirken, puerperal mastit genellikle antibiyotik tedavisiyle iyileşir.

Her iki durumun da doğru teşhis ve tedavi için bir sağlık profesyoneli tarafından değerlendirilmesi önemlidir. Granülomatöz mastit ve puerperal mastit arasındaki farkları ayırt edebilmek için kapsamlı bir değerlendirme ve uygun testler gereklidir.

**You said:**

9) Puerperal mastit nasıl tedavi edilir?

**ChatGPT said:**

ChatGPT

Puerperal mastit tedavisi, iltihabın şiddetine ve varsa komplikasyonlara bağlı olarak değişir. İşte puerperal mastitin genel tedavi yöntemleri:

**1. Antibiyotikler**

- **Amaç:** Bakteriyel enfeksiyonları tedavi etmek ve iltihabı kontrol altına almak.
- **Tedavi:** Genellikle doktorlar, mastitin sebep olduğu bakterilere karşı etkili olan geniş spektrumlu antibiyotikler reçete eder. İlaçlar genellikle oral yolla alınır ve tedavi süresi genellikle 10-14 gün olabilir.

**2. Sıcak Kompresler**

- **Amaç:** Meme üzerindeki iltihaplanmayı azaltmak ve ağrıyı hafifletmek.
- **Uygulama:** Gün boyunca birkaç kez sıcak kompres uygulamak, iltihaplı bölgedeki ağrı ve şişliği hafifletebilir. Kompresler, meme üzerinde birkaç dakika tutulur.

**3. Ağrı Kesiciler**

- **Amaç:** Ağrıyı ve rahatsızlığı hafifletmek.
- **Tedavi:** Doktorlar genellikle asetaminofen (parasetamol) veya ibuprofen gibi reçetesiz ağrı kesiciler önerir. Bu ilaçlar hem ağrıyı hafifletir hem de iltihaplanmayı azaltabilir.

**4. Emzirme veya Süt Pompalama**

- **Amaç:** Sütün düzenli olarak boşaltılmasını sağlamak ve süt birikimini önlemek.
- **Uygulama:** Mastit tedavisinde düzenli emzirme veya süt pompalama çok önemlidir. Bebeğin memeyi etkili bir şekilde emmesi veya süt pompası kullanarak sütün boşaltılması, süt birikimini ve tıkanıklıkları azaltabilir.

**5. Meme Masajı**

- **Amaç:** Süt akışını teşvik etmek ve tıkanıklıkları azaltmak.
- **Uygulama:** Meme üzerinde nazikçe yapılan masaj, süt akışını iyileştirebilir ve tıkanıklıkları hafifletebilir. Masaj yaparken dikkatli olunmalı ve iltihaplı bölgeye zarar vermemeye özen gösterilmelidir.

**6. Cerrahi Müdahale (Gerekirse)**

- **Amaç:** Abse oluşumu durumunda irin boşaltmak.
- **Tedavi:** Eğer mastit, bir abse oluşturmuşsa ve ilaç tedavisi ile iyileşmiyorsa, cerrahi müdahale gerekebilir. Abse, lokal anestezi altında boşaltılabilir ve irin drenajı yapılabilir.

**7. Meme Hijyeni**

- **Amaç:** Enfeksiyon riskini azaltmak.
- **Uygulama:** Meme hijyenine dikkat edilmesi, enfeksiyon riskini azaltabilir. Meme ucu temizliği ve uygun emzirme teknikleri önemlidir. Ayrıca, meme ucu çatlakları veya yaraları önlemek için uygun bakım yapılmalıdır.

**Tedavi Sonrası İzleme**

- **Amaç:** İyileşmeyi değerlendirmek ve komplikasyonları önlemek.
- **Uygulama:** Tedavi sonrası doktor düzenli kontroller yaparak iyileşmeyi değerlendirir ve gerekli durumlarda tedavi planında değişiklikler yapar.

Puerperal mastit tedavisinde, erken müdahale ve uygun tedavi yöntemlerinin uygulanması iyileşme sürecini hızlandırabilir ve komplikasyon riskini azaltabilir. Şiddetli vakalarda veya tedaviye yanıt vermeyen durumlarda, bir sağlık profesyoneline danışmak önemlidir.

**You said:**

10) Antibiyotik tedavisi puerperal mastit için her zaman gerekli midir?

**ChatGPT said:**

ChatGPT

Antibiyotik tedavisi puerperal mastit için genellikle gerekli olmakla birlikte, her durum için aynı şekilde uygulanmayabilir. Antibiyotiklerin gerekli olup olmadığı ve hangi tür antibiyotiklerin kullanılacağı, mastitin şiddetine ve diğer faktörlere bağlıdır. İşte antibiyotik tedavisinin gerekliliği ve koşulları hakkında detaylar:

****1. Bakteriyel Enfeksiyonun Varlığı**

- **Gerekli Durum:** Puerperal mastit, genellikle bakteriyel enfeksiyon sonucu oluşur. Enfeksiyonun tedavi edilmesi için antibiyotikler çoğu zaman gereklidir.
- **Belirtiler:** Meme üzerinde ağrı, şişlik, kızarıklık, ateş ve sistemik belirtiler (titreme, halsizlik) gibi belirtiler varsa, bakteriyel bir enfeksiyon olasılığı yüksektir ve antibiyotik tedavisi genellikle tavsiye edilir.

****2. Tedavi Yanıtı ve Şiddet**

- **Hafif Vakalar:** Hafif mastit vakalarında, meme hijyeni, düzenli emzirme veya süt pompalama ve sıcak kompresler gibi konservatif yöntemler ilk olarak denenebilir. Bu durumlarda, antibiyotik tedavisi hemen başlamayabilir.
- **Şiddetli Vakalar:** Şiddetli mastit veya komplikasyon (örneğin, abse oluşumu) durumlarında, antibiyotik tedavisi hemen başlanmalıdır. Antibiyotikler enfeksiyonun yayılmasını önlemeye ve iyileşmeyi hızlandırmaya yardımcı olabilir.

****3. Süt Kültürü ve Antibiyogram**

- **Kültür ve Hassasiyet:** Bazen süt kültürü yapılabilir ve antibiyogram ile bakteriyel patojenin hangi antibiyotiklere duyarlı olduğu belirlenebilir. Bu bilgi, uygun antibiyotik seçimini yapmada yardımcı olabilir.
- **Genel Tedavi:** Eğer süt kültürü yapılmazsa, doktorlar genellikle geniş spektrumlu antibiyotikler reçete eder. Bu antibiyotikler çoğu yaygın mastit bakterisine karşı etkili olabilir.

****4. Alternatif Tedavi Yöntemleri**

- **Konservatif Yöntemler:** Bazı hafif mastit vakalarında antibiyotik tedavisi yerine konservatif yöntemler tercih edilebilir. Ancak, bu yöntemler genellikle antibiyotik tedavisi ile birlikte veya öncesinde uygulanır.
- **İzleme ve Değerlendirme:** Eğer antibiyotik tedavisi verilmezse veya gecikirse, tedavi süreci ve iyileşme durumu dikkatlice izlenmelidir. Belirtiler kötüleşirse veya iyileşme sağlanmazsa, antibiyotik tedavisi düşünülmelidir.

**Özet**

Antibiyotik tedavisi puerperal mastit için genellikle gereklidir, özellikle bakteriyel enfeksiyonların olduğu durumlarda. Ancak, tedavi yaklaşımı mastitin şiddetine, belirtilere ve tedaviye yanıt durumuna bağlı olarak değişebilir. Doktorlar, hastanın durumunu değerlendirerek en uygun tedavi planını belirleyecek ve gerekirse antibiyotik tedavisine karar verecektir.

**You said:**

11) Puerperal mastit olan bir anne emzirmeye devam edebilir mi?

**ChatGPT said:**

ChatGPT

Puerperal mastit olan bir anne genellikle emzirmeye devam edebilir, ancak bazı durumlarda dikkatli bir yaklaşım gerekebilir. Emzirmeye devam etmek, süt akışının devamını sağlamak ve mastitin iyileşme sürecini desteklemek açısından önemlidir. İşte puerperal mastit durumunda emzirme ile ilgili bazı önemli noktalar:

****1. Emzirmeye Devam Etme**

- **Öneri:** Mastit sırasında emzirmeye devam etmek genellikle önerilir. Emzirme, süt birikimini önlemeye ve süt akışını teşvik etmeye yardımcı olabilir, bu da iltihaplanmanın ve tıkanıklıkların azalmasına yardımcı olabilir.
- **Teknikler:** Bebeğin memeyi etkili bir şekilde emmesi önemlidir. Bebeğin doğru emme tekniği ile memeyi boşaltması, süt kanallarındaki tıkanıklıkları azaltabilir ve mastitin iyileşmesine yardımcı olabilir.

****2. Süt Pompalama**

- **Alternatif:** Eğer emzirmek zor veya ağrılı ise, süt pompası kullanarak süt boşaltılabilir. Süt pompalama, memedeki süt birikimini ve tıkanıklıkları önlemeye yardımcı olabilir.
- **Sık Sık Pompalama:** Süt pompalama işleminin sık sık yapılması, süt akışını korur ve mastitin ilerlemesini önleyebilir.

****3. Ağrı ve Rahatsızlık Yönetimi**

- **Ağrı Yönetimi:** Mastit nedeniyle emzirme sırasında ağrı veya rahatsızlık hissedilirse, uygun ağrı kesiciler ve sıcak kompresler kullanarak rahatlama sağlanabilir.
- **Pozisyon:** Emzirme sırasında rahat bir pozisyon seçmek ve meme üzerinde baskıyı azaltmak da faydalı olabilir.

****4. Hijyen ve Temizlik**

- **Meme Hijyeni:** Meme ucu temizliğine dikkat edilmelidir. Emzirme öncesi ve sonrası ellerin temiz olması ve meme ucu bölgesinin temiz tutulması enfeksiyon riskini azaltabilir.
- **Meme Bakımı:** Meme ucu çatlaklarını önlemek için uygun bakım ve kremler kullanılabilir.

****5. Antibiyotik Kullanımı**

- **Emzirme ve İlaçlar:** Eğer antibiyotik tedavisi gerekiyorsa, doktorlar genellikle emzirme sırasında güvenli olan antibiyotikler reçete eder. Bu antibiyotikler genellikle bebek üzerinde olumsuz etki yapmaz.

****6. Komplikasyonlar ve İzleme**

- **Belirtiler:** Eğer mastit belirtileri kötüleşirse veya abse oluşumu gibi komplikasyonlar gelişirse, doktorla iletişime geçmek ve uygun tedavi almak önemlidir.
- **İzleme:** Mastitin tedavi süreci ve iyileşme durumunu izlemek önemlidir. Doktorlar, emzirmeye devam etme ve diğer tedavi seçenekleri konusunda rehberlik edebilir.

**Özet**

Puerperal mastit olan bir anne emzirmeye devam edebilir, hatta bu, tedavi sürecinin bir parçası olarak önerilir. Emzirme, süt akışını destekler ve iltihaplanmanın azaltılmasına yardımcı olabilir. Ancak, emzirmeye devam etme sürecinde ağrı, hijyen ve tedavi gibi faktörlere dikkat edilmelidir. Her durumda, anne ve bebeğin sağlığını korumak için bir sağlık profesyoneli ile düzenli olarak iletişimde olmak en iyisidir.

**You said:**

12) Puerperal mastitin tekrarlama riski var mı?

**ChatGPT said:**

ChatGPT

Evet, puerperal mastitin tekrarlama riski vardır. Mastit tedavi edildikten sonra bile, bazı kadınlar tekrar mastit geliştirebilir. Tekrarlama riskini etkileyen faktörler ve önlemler hakkında daha fazla bilgi:

**Tekrarlama Riskini Artıran Faktörler**

1. **Süt Kanallarında Tıkanıklıklar:**
   - Süt kanallarında tıkanıklıklar veya süt birikimi mastit riskini artırabilir. Süt birikimi, özellikle emzirme veya süt pompalama tekniklerinin yetersiz olduğu durumlarda ortaya çıkabilir.
2. **Meme Ucu Çatlakları veya Yaralar:**
   - Meme ucu çatlakları veya yaraları bakteriyel enfeksiyonlar için giriş yolu sağlayabilir. Bu tür çatlaklar mastit riskini artırabilir.
3. **Yetersiz Emzirme veya Süt Boşaltımı:**
   - Bebeklerin memeyi yeterince etkili bir şekilde emmemesi veya süt pompalama sıklığının yetersiz olması, süt birikimini ve dolayısıyla mastit riskini artırabilir.
4. **Hijyen Sorunları:**
   - Meme hijyenine dikkat edilmemesi, enfeksiyon riskini artırabilir. Meme ucu bölgesinin temizliği ve bakımına dikkat edilmelidir.
5. **Bağışıklık Sistemi Sorunları:**
   - Bağışıklık sistemi zayıflamış olan kadınlar veya diabet gibi sağlık sorunları yaşayanlar mastit riski taşıyabilir.
6. **Sık Tekrarlayan Enfeksiyonlar:**
   - Önceki mastit vakaları veya diğer enfeksiyonlar, tekrarlama riskini artırabilir.

**Önlemler ve Yönetim**

1. **Emzirme Teknikleri:**
   - Bebeklerin memeyi etkili bir şekilde emmesi sağlanmalı ve süt pompalama yöntemleri doğru uygulanmalıdır. Düzenli ve etkili emzirme, süt birikimini ve tıkanıklıkları önlemeye yardımcı olabilir.
2. **Meme Hijyeni:**
   - Meme hijyenine özen gösterilmeli, meme ucu çatlaklarından kaçınılmalı ve gerektiğinde uygun bakım yapılmalıdır.
3. **Sık Sık Emzirme veya Pompalama:**
   - Süt akışını desteklemek için sık sık emzirme veya süt pompalama uygulanmalıdır. Bu, süt kanallarındaki tıkanıklıkları önlemeye yardımcı olabilir.
4. **Meme Bakımı:**
   - Meme ucu çatlaklarını ve yaralarını önlemek için uygun bakım ve kremler kullanılabilir.
5. **Doktor Kontrolleri:**
   - Eğer mastit belirtileri tekrar ederse veya yeni belirtiler gelişirse, bir sağlık profesyoneline başvurmak önemlidir. Erken müdahale ve tedavi, komplikasyon riskini azaltabilir.
6. **Sağlık Sorunları Yönetimi:**
   - Diğer sağlık sorunları veya bağışıklık sistemi ile ilgili sorunlar varsa, bunlar da yönetilmelidir.

**Özet**

Puerperal mastitin tekrarlama riski vardır ve bu risk, süt kanalı tıkanıklıkları, meme ucu çatlakları, yetersiz emzirme, hijyen sorunları ve bağışıklık sistemi sorunları gibi faktörlerden kaynaklanabilir. Tekrar riskini azaltmak için uygun emzirme tekniklerine, meme hijyenine ve düzenli kontrol ve bakımına dikkat edilmelidir. Eğer mastit belirtileri tekrar ederse, bir sağlık profesyoneli ile görüşmek ve uygun tedavi yöntemlerini uygulamak önemlidir.

**You said:**

13) Puerperal mastit uzun vadede hangi komplikasyonlara yol açabilir?

**ChatGPT said:**

ChatGPT

Puerperal mastit, tedavi edilmediğinde veya yetersiz tedavi edildiğinde bazı uzun vadeli komplikasyonlara yol açabilir. İşte puerperal mastitin uzun vadeli potansiyel komplikasyonları:

**1. Abse Oluşumu**

- **Tanım:** Mastitin ilerlemesi sonucunda, iltihaplı bölgede irin birikerek bir abse (irin dolu kitle) oluşabilir.
- **Komplikasyon:** Abse, genellikle cerrahi drenaj gerektirir. İyi tedavi edilmezse, abse tekrarlayabilir ve ciddi sağlık sorunlarına yol açabilir.

**2. Kronik Mastit**

- **Tanım:** Akut mastitin tekrarlayan veya tedavi edilmemiş formlarında, iltihaplanma kronik hale gelebilir.
- **Komplikasyon:** Kronik mastit, uzun süreli ağrı, şişlik ve iltihaplanma ile karakterize olabilir. Tedavi edilmediğinde kalıcı meme hasarına yol açabilir.

**3. Meme Dokusunda Hasar**

- **Tanım:** Uzun süreli iltihaplanma ve enfeksiyonlar meme dokusunda kalıcı hasara neden olabilir.
- **Komplikasyon:** Bu hasar, meme dokusunun sertleşmesine, şekil bozukluklarına veya elastikiyet kaybına yol açabilir. Ayrıca, bazı durumlarda süt kanallarında kalıcı tıkanıklıklar oluşabilir.

**4. Süt Kanalı Tıkanıklıkları ve Dolaşım Problemleri**

- **Tanım:** Süt kanallarında uzun süreli tıkanıklıklar ve iltihaplanma, normal süt akışını etkileyebilir.
- **Komplikasyon:** Tıkanıklıklar, süt birikimine ve dolayısıyla süt akışında düzensizliklere yol açabilir. Bu durum, emzirme sorunlarına ve süt üretiminde azalmaya neden olabilir.

**5. Psikolojik Etkiler**

- **Tanım:** Kronik ağrı, tekrarlayan enfeksiyonlar ve emzirme sorunları, annenin ruh sağlığını etkileyebilir.
- **Komplikasyon:** Depresyon, anksiyete ve stres gibi psikolojik problemler gelişebilir. Annenin genel iyilik hali ve ebeveynlik deneyimi üzerinde olumsuz etkiler olabilir.

**6. Emzirme Sorunları**

- **Tanım:** Mastit nedeniyle memede ağrı, şişlik veya iltihaplanma emzirme sürecini zorlaştırabilir.
- **Komplikasyon:** Emzirme sırasında rahatsızlık, bebeğin memeyi yeterince etkili bir şekilde emmemesine ve dolayısıyla süt üretiminin azalmasına yol açabilir. Bu da annenin emzirmeyi sürdürmesini zorlaştırabilir.

**7. İltihaplı Kitleler veya Kalsifikasyonlar**

- **Tanım:** Uzun süreli iltihaplanma, meme dokusunda kalsifikasyonlara veya iltihaplı kitlelere neden olabilir.
- **Komplikasyon:** Bu kitleler, meme muayenelerinde veya görüntüleme testlerinde anormal bulgulara yol açabilir ve ek değerlendirmeler gerektirebilir.

**Önleme ve İzleme**

- **Erken Tedavi:** Mastitin erken teşhisi ve uygun tedavisi, komplikasyon riskini azaltabilir.
- **Düzenli Kontroller:** Annenin mastit tedavi sürecinde düzenli doktor kontrolleri ve izleme, olası komplikasyonların erken tespiti için önemlidir.
- **Emzirme Desteği:** Emzirme teknikleri, meme hijyeni ve bakım konularında profesyonel destek almak, komplikasyon riskini azaltabilir.

**Özet**

Puerperal mastit, tedavi edilmediğinde veya yetersiz tedavi edildiğinde uzun vadeli komplikasyonlara yol açabilir. Bu komplikasyonlar arasında abse oluşumu, kronik mastit, meme dokusunda hasar, süt kanalında tıkanıklıklar, psikolojik etkiler ve emzirme sorunları bulunur. Erken ve uygun tedavi ile komplikasyon riski azaltılabilir ve uzun vadeli sağlık sorunlarının önüne geçilebilir.

**You said:**

14)Mastit tedavisinden sonra memede sertlik kalır mı?

**ChatGPT said:**

ChatGPT

Mastit tedavisinden sonra memede sertlik kalıp kalmayacağı, tedavi sürecinin ne kadar etkili olduğuna, mastitin şiddetine ve bireysel iyileşme süreçlerine bağlı olarak değişir. İşte mastit tedavisinden sonra memede kalabilecek sertliklerle ilgili bazı genel bilgiler:

****1. Geçici Sertlik**

- **Tanım:** Mastit tedavisinin ardından, bazı durumlarda memede geçici sertlik veya şişlik kalabilir.
- **Nedenler:** Bu, iltihaplanma sürecinden kaynaklanan geçici ödem veya iyileşme sürecinin bir parçası olabilir. Süt kanallarındaki tıkanıklıkların düzelmesi ve şişliklerin azalması zaman alabilir.

****2. Kronik Sertlik veya Doku Hasarı**

- **Tanım:** Uzun süreli veya şiddetli mastit vakalarında, tedavi sonrası kalıcı sertlik veya meme dokusunda değişiklikler meydana gelebilir.
- **Nedenler:** İltihap ve enfeksiyon süreci meme dokusunda kalıcı hasara neden olabilir. Bu, meme dokusunun sertleşmesine veya kalsifikasyonlara yol açabilir.

****3. Süt Kanalı Tıkanıklıkları**

- **Tanım:** Mastit sonrasında bazı süt kanallarında kalıcı tıkanıklıklar veya düzensizlikler oluşabilir.
- **Nedenler:** Tıkanıklıklar, meme dokusunda sertlik ve kitleler oluşturabilir. Bu, özellikle süt kanallarında uzun süreli iltihaplanma yaşayan kadınlarda görülebilir.

****4. Emzirme ve Süt Boşaltımı**

- **Tanım:** Emzirme veya süt boşaltımı sırasında memede kalabilecek sertlikler, süt birikimi ve tıkanıklıkların bir sonucu olabilir.
- **Nedenler:** Süt akışının düzelmesi ve memedeki süt birikiminin azaltılması ile sertlik genellikle zamanla azalır. Ancak, bazı durumlarda tedavi sürecinde kalıcı değişiklikler meydana gelebilir.

****5. İzleme ve Değerlendirme**

- **Tedavi Sonrası:** Mastit tedavisinden sonra, memede kalıcı sertlik veya diğer anormal bulgular varsa, bir sağlık profesyoneline danışmak önemlidir. Doktor, sertliğin nedenini değerlendirecek ve gerekli testleri yaparak uygun tedavi önerilerinde bulunacaktır.
- **Görüntüleme Testleri:** Kalıcı sertlik veya anormal dokular varsa, meme ultrasonu veya diğer görüntüleme testleri ile detaylı bir değerlendirme yapılabilir.

**Önleme ve Yönetim**

- **Düzenli Kontroller:** Mastit tedavisinden sonra düzenli doktor kontrolleri, memede kalıcı sertliklerin veya diğer komplikasyonların erken teşhis edilmesine yardımcı olabilir.
- **Meme Bakımı:** Meme hijyenine dikkat etmek, emzirme tekniklerini doğru uygulamak ve gerektiğinde profesyonel destek almak, iyileşme sürecini destekleyebilir.

**Özet**

Mastit tedavisinden sonra memede geçici veya kalıcı sertlikler olabilir. Geçici sertlik genellikle iyileşme sürecinin bir parçası olarak düzelir, ancak kalıcı sertlik veya doku değişiklikleri, mastitin şiddetine ve tedaviye yanıtına bağlı olarak gelişebilir. Eğer mastit tedavisinden sonra memede sertlik veya diğer anormal bulgular varsa, bir sağlık profesyoneline danışmak ve uygun izleme yapmak önemlidir.

**You said:**

15)Mastit oluşumunu önlemek için neler yapılmalıdır?

**ChatGPT said:**

ChatGPT

Mastit oluşumunu önlemek için çeşitli stratejiler uygulanabilir. Bu stratejiler, özellikle emzirme döneminde meme sağlığını korumak ve süt akışını düzenlemek üzerine odaklanır. İşte mastit oluşumunu önlemek için alınabilecek önlemler:

****1. Doğru Emzirme Teknikleri**

- **Etkili Emzirme:** Bebeğin memeyi etkili bir şekilde emmesini sağlamak, süt birikimini ve tıkanıklıkları önlemeye yardımcı olabilir. Bebeğin ağız pozisyonu ve emme tekniği önemlidir.
- **Sık Sık Emzirme:** Bebeği düzenli olarak ve sık sık emzirmek, süt kanallarında tıkanıklık ve birikimi önleyebilir. Genellikle her 2-3 saatte bir emzirme önerilir.

****2. Süt Boşaltımı ve Pompalama**

- **Süt Boşaltımı:** Bebeğin emziremeyeceği durumlarda veya emzirme sıklığının yetersiz olduğu durumlarda süt pompası kullanarak süt boşaltılabilir. Sütün düzenli olarak boşaltılması, tıkanıklıkları ve süt birikimini önlemeye yardımcı olabilir.
- **Pompa Hijyeni:** Süt pompası ve aksesuarlarının temizliği de önemlidir. Temiz olmayan pompa ekipmanları bakteriyel enfeksiyon riskini artırabilir.

****3. Meme Hijyeni ve Bakımı**

- **Temizlik:** Meme ucu bölgesinin temizliği önemlidir. Ancak, aşırı temizleme veya sert sabun kullanımı meme ucunun kurumasına ve çatlamasına yol açabilir. Hafif sabun ve su kullanarak nazikçe temizlemek yeterlidir.
- **Çatlakların Önlenmesi:** Meme ucu çatlaklarını önlemek için uygun emzirme teknikleri ve nemlendiriciler kullanılabilir. Çatlaklar bakteriyel enfeksiyonlar için giriş yolu sağlayabilir.

****4. Rahatsızlıkları Azaltma**

- **Rahat Pozisyonlar:** Emzirme sırasında rahat pozisyonlar seçmek, meme üzerindeki baskıyı azaltabilir ve ağrıyı hafifletebilir.
- **Sıcak Kompresler:** Emzirme öncesi sıcak kompresler kullanmak, süt akışını teşvik edebilir ve tıkanıklıkları önleyebilir.

****5. Süt Kanalı Tıkanıklıklarının Önlenmesi**

- **Sıkı Giysilerden Kaçınma:** Meme üzerinde baskı yapabilecek sıkı giysiler veya sutyenlerden kaçınılmalıdır. Sıkı giysiler, süt kanallarının tıkanmasına yol açabilir.
- **Meme Masajı:** Emzirme veya süt pompalama öncesi nazikçe yapılan masaj, süt akışını teşvik edebilir ve tıkanıklıkları azaltabilir.

****6. Sağlık Durumunun Yönetimi**

- **Genel Sağlık:** Genel sağlık durumunu yönetmek ve bağışıklık sistemini güçlendirmek, enfeksiyonlara karşı direnç sağlayabilir.
- **Diyet ve Sıvı Alımı:** Yeterli sıvı alımı ve dengeli bir diyet, vücudun iyileşme ve enfeksiyonlarla savaşma kapasitesini destekleyebilir.

****7. Eğitim ve Destek**

- **Eğitim:** Yeni annelere emzirme teknikleri ve meme bakımı hakkında eğitim verilmesi, mastit riskini azaltabilir.
- **Destek:** Emzirme danışmanları ve sağlık profesyonellerinden destek almak, emzirme sürecini optimize edebilir ve sorunları erkenden tespit edebilir.

****8. Düzenli Kontroller**

- **Sağlık Kontrolleri:** Düzenli doktor kontrolleri, meme sağlığını izlemek ve olası sorunları erken teşhis etmek için önemlidir. Mastit belirtileri fark edildiğinde erken müdahale, komplikasyon riskini azaltabilir.

**Özet**

Mastit oluşumunu önlemek için doğru emzirme tekniklerine, süt boşaltımına, meme hijyenine ve rahatsızlıkları azaltmaya yönelik önlemler almak önemlidir. Ayrıca, meme üzerindeki baskıyı azaltmak, genel sağlık durumunu yönetmek ve profesyonel destek almak, mastit riskini azaltabilir. Düzenli kontroller ve eğitim de önemlidir, böylece herhangi bir problem erken tespit edilip gerekli önlemler alınabilir.
