# Supplementary material for: Evaluation of ChatGPT-4 as an Online Outpatient Assistant in Puerperal Mastitis Management: Content Analysis of an Observational Study
Source: JMIR Med Inform. 2025 Jul 24;13:e68980. doi: 10.2196/68980 (PMC12288767; doi:10.2196/68980)
Supplement: Multimedia Appendix 4 [file medinform-v13-e68980-s004.docx]

**Appendix 4: Distribution of the average scores of the evaluators based on questions**

| Question Number | Sufficient length | Understandability | Accuracy | Compliance with literature | Patient safety | total |
| --- | --- | --- | --- | --- | --- | --- |
|  | mean±sd (M) | mean±sd (M) | mean±sd (M) | mean±sd (M) | mean±sd (M) | mean±sd (M) |
| 1 | 4,4±0,89 (5) | 5±0 (5) | 4,6±0,55 (5) | 4±0,71 (4) | 4,4±0,55 (4) | 22,4±1,82 (22) |
| 2 | 4,6±0,55 (5) | 4,8±0,45 (5) | 4,6±0,55 (5) | 4,2±0,84 (4) | 4,8±0,45 (5) | 23±2 (23) |
| 3 | 4,8±0,45 (5) | 5±0 (5) | 4,6±0,55 (5) | 4,4±0,89 (5) | 4,8±0,45 (5) | 23,6±1,95 (25) |
| 4 | 4±0 (4) | 4,4±0,55 (4) | 3,8±0,84 (4) | 3,6±1,14 (4) | 4,4±0,55 (4) | 20,2±2,77 (21) |
| 5 | 4,6±0,55 (5) | 5±0 (5) | 4,8±0,45 (5) | 4,6±0,55 (5) | 4,6±0,55 (5) | 23,6±1,52 (24) |
| 6 | 4,8±0,45 (5) | 4,8±0,45 (5) | 4,6±0,55 (5) | 4,2±0,84 (4) | 4,6±0,55 (5) | 23±2,55 (24) |
| 7 | 4,2±0,45 (4) | 5±0 (5) | 4,6±0,55 (5) | 4,4±0,89 (5) | 4,8±0,45 (5) | 23±1,87 (24) |
| 8 | 4,6±0,55 (5) | 4,4±0,55 (4) | 4,2±0,84 (4) | 4,2±1,3 (5) | 4,6±0,55 (5) | 22±3,46 (23) |
| 9 | 4,6±0,55 (5) | 4,8±0,45 (5) | 4,6±0,55 (5) | 4,6±0,55 (5) | 4,8±0,45 (5) | 23,4±1,82 (24) |
| 10 | 4,6±0,55 (5) | 4,8±0,45 (5) | 4,6±0,55 (5) | 4,4±0,55 (4) | 4,8±0,45 (5) | 23,2±1,48 (23) |
| 11 | 4,4±0,55 (4) | 4,8±0,45 (5) | 4,4±0,89 (5) | 4±1 (4) | 4,6±0,89 (5) | 22,2±2,95 (22) |
| 12 | 5±0 (5) | 5±0 (5) | 4,6±0,55 (5) | 4,2±0,84 (4) | 4,6±0,55 (5) | 23,4±1,52 (23) |
| 13 | 4,4±0,55 (4) | 4,8±0,45 (5) | 4,4±0,89 (5) | 4,2±1,1 (5) | 4,4±0,55 (4) | 22,2±2,77 (22) |
| 14 | 4,8±0,45 (5) | 5±0 (5) | 4,6±0,55 (5) | 4±0,71 (4) | 4,8±0,45 (5) | 23,2±1,64 (24) |
| 15 | 4,4±0,55 (4) | 4,4±0,55 (4) | 4,6±0,55 (5) | 4,4±0,89 (5) | 4,4±0,55 (5) | 22,2±1,64 (22) |
| p | 0,241 | 0,075 | 0,923 | 0,952 | 0,877 | 0,851 |

The average score per question was 4.54±0.69. The average scores for ChatGPT responses to each query ranged from 3.6±1.14 to 5 (Table 4). Simultaneously, no statistical distinctions were observed between the scores of the questions within the scope of sufficient length, understandable language, accuracy, compliance with the literature, and Patient Safety when evaluated according to the scope of the question(s).
